# Proteins are a source of glycans found in preparations of glycoRNA

**DOI:** 10.1038/s12276-025-01575-1

**Published:** 2025-11-14

**Authors:** Nathanael B. Kegel, Nurseda Yilmaz Demirel, Timo Glatter, Katharina Höfer, Andreas Kaufmann, Stefan Bauer

**Affiliations:** 1 Institute of Immunology, Philipps-Universität Marburg, Marburg, Germany; 2 Max Planck Institute for Terrestrial Microbiology, Marburg, Germany; 3 Center for Synthetic Microbiology, Philipps-Universität Marburg, Marburg, Germany; 4 Department of Pharmacy, Institute of Pharmaceutical Biology and Biotechnology, Philipps-Universität Marburg, Marburg, Germany

**Keywords:** Proteomics, Electrophoresis

## Abstract

Recent discoveries suggesting that RNA can be modified with sialylated glycans (termed glycoRNA) could broaden our understanding of cellular glycosylation beyond traditional proteins and lipids. However, the pathway of RNA-glycosylation and its biological function remain elusive. Following the original glycoRNA isolation protocol, we also detect labeled glycans in small RNA preparations. However, glycosylated molecules showed resistance to treatment with RNase A/T1 but were sensitive to proteinase K digestion under denaturing conditions. Using liquid chromatography-mass spectrometry-based proteomics we here detect various proteins that copurify with small but not large RNA preparations isolated from human or murine cells, including the glycosylated membrane protein LAMP1. Importantly, we further demonstrate that recombinant soluble LAMP1 can be purified following the glycoRNA isolation method. These findings suggest that glycoproteins copurify with RNA using current glycoRNA purification protocols, thus representing a considerable source of glycans in samples of glycoRNA.

## Purified glycoRNA preparations contain glycosylated proteins

Glycosylation, a process where sugars attach to molecules, is known to modify proteins and lipids. Recently, researchers discovered that RNA can also be glycosylated. This study explores the presence of glycoproteins in RNA samples, which might affect the study of glycoRNA. Researchers used a method involving metabolic labeling and silica columns to study glycoRNA. They found that glycoproteins, such as LAMP1, copurify with RNA, meaning that they are extracted together during the purification process. This copurification is influenced by the presence of intact RNA and alcohol concentration. The study concludes that glycoproteins can mimic glycoRNA owing to their similar properties, potentially complicating research. Future studies should refine methods to separate these molecules more effectively, ensuring the accurate analysis of glycoRNA. This could improve our understanding of RNA modifications and their roles in health and disease. This summary was initially drafted using artificial intelligence, then revised and fact-checked by the author.

## Introduction

Glycosylation shapes the structure, interactions and signaling properties of its targets^1,2^, which traditionally are considered to be proteins and lipids. With the exception of monosaccharide attachment to the noncanonical tRNA nucleoside queuosine^3–5^, RNA was not known to be a target of glycosylation, until Flynn et al. reported N-glycosylation as the newest type of RNA modification in living cells^6^. The group used metabolic labeling with peracetylated *N*-azidoacetylmannosamine (Ac_4_ManNAz) to introduce a clickable azido-sialic acid to nascent N-glycans^7^, thus enabling the attachment of chemical reporters such as biotin to the labeled glycans by strain-promoted azide–alkyne cycloaddition^8^. By using a streptavidin probe, labeled glycans were detected in RNA samples via northern blotting. The signals displayed high molecular weight in the range of >10 kb and were lost upon the disruption of the N-glycosylation machinery and upon RNase A/T1 treatment, yet remained unaffected by DNase I or proteinase K. Despite the high apparent molecular weight, the glycan incorporation was restricted to preparations of small RNAs of less than 200 bp. RNA sequencing of affinity-purified glycoconjugates identified small noncoding RNAs, such as Y, U, small nucleolar and transfer RNAs, as possible targets of RNA glycosylation. Interestingly, subcellular fractionation and subsequent RNA extraction demonstrated that glycan-modified RNAs are associated to the outside of cellular membranes, where they serve as ligands for sialic acid-binding lectins 11 and 14. Together, these data provide evidence for the existence of a cell-surface-attached covalently linked RNA–glycan conjugate (hereafter: glycoRNA)^6^. The discovery of glycoRNAs has raised questions regarding the nature of the RNA–glycan linkage, the underlying pathway by which sugar chains are attached to RNA and the biological function^9–12^. Recently, leveraging a periodate-based method for the detection of glycans in native RNA, the noncanonical RNA base 3-(3-amino-3-carboxypropyl)uridine (acp^3^U) was reported as the N-glycan attachment site on RNA^13^. Studies exploring the functional aspects of this putative modification report a multifaceted role in both homeostatic and disease-related processes. Recently, glycoRNA was observed in the contexts of tumor progression and monocyte adhesion to endothelial cells^14^, in neutrophil recruitment to inflammatory sites^15^ and in the regulation of epithelial barrier function in the lung^16^. Moreover, tight interactions of glycoRNA with other components of the cell membrane, such as lipid rafts, cell-surface proteins and heparan sulfate^14,17–19^ indicate the existence of complex microenvironments at the cell surface.

Even though the glycoRNA field is expanding with new discoveries, a current study reported that RNA preparations contain N-glycosylated species whose sensitivity to RNase treatment depends on specific purification steps^20^. The existence of such artifacts could stymie attempts to study this novel RNA modification using current assays and weaken evidence used to support conclusions in previous studies. In line with this finding, we here show that the reported elimination of detectable glycans from RNA by RNase treatment depends on a postdigestion silica column purification step. We observed that the column binding of the labeled glycosylated molecules is impaired upon RNase treatment, and labeled glycans can be recovered on the basis of the purification steps. Analysis of glycan-rich RNA by sodium dodecyl sulfate–polyacrylamide gel electrophoresis (SDS–PAGE) revealed the presence of a glycosylated molecule of approximately 100 kDa. Importantly, we show that this glycomolecule is sensitive to proteinase K digestion under denaturing conditions. Mass spectrometry-based proteomics confirmed that several glycoproteins, among them LAMP1, copurify with small RNA preparations and withstand the extraction strategy described in the glycoRNA protocol^6^. Our findings suggest that glycoproteins contribute to the level of detectable glycans in RNA preparations.

## Material and methods

### Cells

NIH3T3 cells (3T3 cells) were grown in DMEM (Dulbecco’s modified Eagle medium) (Anprotec, AC-LM0015) with 10% (vol/vol) fetal calf serum (Gibco), 2 mM of ʟ-glutamine (Gibco) and 100 IU/ml of penicillin–streptomycin (Gibco) at 37 °C, 7.5% CO_2_. HeLa cells were cultivated in RPMI (Roswell Park Memorial Institute 1640 medium) (Anprotec, AC-LM0051) with identical supplements at 37 °C, 5% CO_2_. The cells were passaged twice per week in a 1:10 ratio in T25 plastic flasks.

### Metabolic labeling

N-glycans were metabolically labeled as described previously^6^. In brief, confluent 3T3 or HeLa cells were collected by trypsin detachment. The cells were pelleted, resuspended in complete cell culture medium and counted using a Neubauer cell counting chamber. Typically, 4.9 × 10^6^ cells were seeded in a T175 cell culture flask. The labeling medium consisted of 25 ml of Dulbecco’s modified Eagle medium or Roswell Park Memorial Institute 1640 medium with supplements as stated above and 100 µM Ac_4_ManNAz (Jena Bioscience), 100 µM GalNAc (Sigma) and 10 µM D-Gal (Roth). Cells were incubated at 37 °C, 7.5% CO_2_ for 40 h.

### RNA extraction

After 40 h of metabolic glycan labeling, the culture medium was aspirated and adherent cells were washed once with phosphate-buffered saline (PBS). TRIzol RNA extraction reagent (Invitrogen, 15596018) was added directly to the cells and incubated for 10 min at ambient temperature. For T175 flasks, 10 ml of TRIzol was applied. The cells were homogenized by pipetting, transferred to a 15-ml reaction tube and incubated for another 10 min at 37 °C to increase lysis efficiency. To initiate phase separation, 0.2× volumes of chloroform (100%) were added and mixed by thorough shaking. After centrifugation at 4,000*g* for 10 min, the aqeuous phase was transferred to a fresh reaction tube and mixed with 1.1× volumes of isopropanol (100%). RNA was precipitated at −20 °C for 1 h, then pelleted by centrifugation at 4,000*g*, 4 °C for 2 h. The pellet was washed once with ethanol (80%), then air-dried using a laminar flow hood and lastly solubilized overnight in ultrapure water (UltraPure, Invitrogen).

### Silica column purifications

Silica columns were used to desalt RNA after the TRIzol extraction and to remove metabolites and unconjugated click chemistry reagents following the adaptions described recently^6,21^. In brief, samples were mixed with two volumes of RNA binding buffer (Zymo Research, R1013-2) and briefly vortexed. An additional volume of isopropanol (100%) was added (for example, 100 µl of RNA + 200 µl RNA binding buffer + 300 µl isopropanol), and the samples were mixed by vortexting and briefly incubated on ice. The samples were loaded to Zymo Spin silica columns and centrifuged for 30 s at 16,000*g*. For up to 70 µg of RNA, Zymo Spin IC columns (Zymo Research, C1004) were used, and IIICG columns (C1006) were used for RNA quantities of up to 350 µg (ref. ^6^). In total, three separate washing steps were included: once with 400 µl RNA Prep Buffer (Zymo Research, R1060-2) and 30 s centrifugation, once with 700 µl ethanol (80%) and 30 s centrifugation and once with 400 µl ethanol (80%) for 60 s to remove residual alcohol. RNA was eluted twice with ultrapure water. After the column purification, RNA concentration was quantified by NanoDrop spectroscopy. Alternatively, when extracting lower amounts of RNA (for example, from six-well plates), we found that precipitation from the aqeuous phase of TRIzol with two volumes of isopropanol (100%) and direct loading to the silica columns led to indentical results. The separation of large (>200 nts) and small (<200 nts) RNAs was conducted according to the protocol described by Hemberger et al.^21^. In brief, total RNA was mixed with two volumes of an adjusted RNA binding buffer (equal parts of RNA binding buffer and 100% ethanol) and loaded to a silica column. After centrifugation, the large RNA fraction was retained in the column, and the flow-through, containing the small RNA fraction, was mixed with an additional volume of isopropanol (100%). The small RNA fraction was loaded to a separate spin column. The purification proceeded as described above.

### Proteinase K treatments

For further RNA sample purification, RNA desalted via silica column purification was treated with proteinase K as described previously^6^. In brief, proteinase K (PCR grade, Thermo Scientific, EO0491) was added directly to RNA in ultrapure water at a concentration of 1 µg proteinase K per 25 µg of RNA. The treatment was performed for 45 min at 37 °C. Alternatively, treatment was conducted in denaturing Tris buffer (DTB) to provoke protein unfolding and enhance enzymatic activity^22^. The DTB was prepared analogous to SDS–PAGE sample buffer^23^ containing 375 mM Tris–HCl (pH 6.8), 9% sodium dodecyl sulfate (SDS), 10% 2-mercaptoethanol, 50% glycerol and 0.003% bromophenol blue. Typically, 25 µg of RNA in 40 µl of ultrapure water was supplemented with 1 µg of proteinase K, then 14 µl of ultrapure water or DTB was added (final buffer concentrations: 97.2 mM Tris–HCl, 2.3% SDS, 2.6% 2-mercaptoethanol, 13% glycerol). After 45 min of treatment, the samples were filled to 100 µl with ultrapure water, and the buffers and metabolites were removed by another silica column clean-up as described above. All RNAs in this study were purified by these steps, except when specified otherwise. To explore proteinase K activity against the glycosylated molecules in preparations of RNA over time, the reaction tubes containing 2 µg of labeled, purified and clicked RNA (see below) in ultrapure water were supplemented with 1 µg of proteinase K per reaction tube and then transferred to a heating block at 37 °C. Next, 0.4× volumes of DTB were added in intervals of 15 min, where the first tube received DTB after 0 min and the last tube received DTB 90 min after the first tube. Typically, 4 µl of DTB was added to 10 µl of the RNA–proteinase K mixture. The samples that received 4 µl of H_2_O instead of DTB were used as a control. After 90 min, 4 µl of DTB was applied to all samples that had not previously received DTB, whereas others received 4 µl of H_2_O to yield a total volume of 18 µl in all samples. The probes were then immediately and simultaneously boiled at 95 °C for 3 min and subsequently analyzed via SDS–PAGE and fluorescence in-gel detection (see below).

### Bioorthogonal click chemistry

The click reactions were performed following the procedure described by Flynn et al.^6^. In short, desalted and proteinase-K-treated RNA was subjected to copper-free click chemistry with 500 µM dibenzocyclooctyne (DBCO)–biotin or DBCO–Alexa Fluor 647 (AF647). Typically, 9 µl of RNA was mixed with 10 µl of dye free gel loading buffer (df-GLB; final concentration: 50% formamide, 9 mM ethylenediaminetetraacetic acid and 0.0125% SDS)^6^ and 1 µl of DBCO–PEG_4_–biotin (Jena Bioscience) or DBCO–AF647 (Jena Bioscience). We found this reaction to be scalable without an evident reduction of signal intensity, and the results were reproducible for RNA inputs between 10 and 300 µg. Conjugation was performed at 55 °C for 10 min. Afterward, samples were filled to 100 µl with ultrapure water, and the excess click reagent was removed by another silica column clean-up as described above.

### Gel electrophoresis, RNA and glycan visualization

Glycan incorporation in RNA preparations was analyzed by fluorescence in-gel detection or by northern blotting. For fluorescence in-gel detection, electrophoresis was performed in agarose gels with 1% agarose (m/vol) and 1.5× 3-(*N*-morpholino)propanesulfonic acid (MOPS) buffer, submerged in 1× MOPS buffer (200 mM MOPS, 50 mM sodium acetate and 10 mM ethylenediaminetetraacetic acid, adjusted to pH 7 with NaOH). Before loading, RNA samples were mixed with 5× denaturing loading buffer (50% formamide, 5.92% formaldehyde, 6.67% glycerol and 1× MOPS buffer) to yield at least 2× buffer concentration. The samples were denatured at 70 °C for 1 min and immediately placed on ice. Electrophoresis was conducted for 45 min at 105 V. The glycans were visualized directly in the agarose gel by fluorescence detection in the AF647 channel. The RNA was visualized by incubating the agarose gel in ethidium bromide solution (2 µg/ml) for 10 min at ambient temperature. The RNA was detected by ultraviolet light using a GelStick Imager (Intas).

For glycan detection by northern blotting, downward alkaline blotting^24^ was used for transfer to a nitrocellulose membrane using a transfer buffer with 3 M NaCl and 8 mM NaOH. The transfer was performed for 150 min at ambient temperature. Afterward, the RNA was immobilized by ultraviolet irradiation (1.23 J/cm^2^), and the membrane was washed twice with PBS for the removal of the transfer buffer. The membrane was blocked overnight in blocking buffer (0.5% bovine serum albumin (BSA) in PBS with 0.1% Tween 20). Streptavidin–horseradish peroxidase (Roche) was diluted 1:5,000 (vol/vol) in blocking buffer and used for the detection of DBCO–biotin-conjugated labeled glycans. The signals were detected with Femto (Thermo Scientific, 34096) and a ChemiDoc MP imaging system. Afterward, total RNA was stained on the membrane with methylene blue^25^. In brief, the nitrocellulose membrane was preincubated in 5% acetic acid for 15 min at ambient temperature. The staining was conducted in 0.5 M sodium acetate (pH 5.2) and 0.04% methylene blue for 10–30 min at ambient temperature until clear ribosomal RNA bands were visible. The membrane was washed several times with desalted water and then photographed using a ChemiDoc MP imager. To probe for the presence of proteins and investigate the protein-like migration of glycan-bearing molecules in preparations of RNA, clicked samples of RNA were analyzed by SDS–PAGE following the procedure described by Laemmli (1970) using gels with 10% polyacrylamide^23^. The RNA samples were mixed with SDS–PAGE sample buffer containing SDS and 2-mercaptoethanol and were immediately boiled at 95 °C for 3 min. The gels were run for at least 2 h until the bromophenol blue front reached the lower gel end, and the glycans were detected by in-gel fluorescence as explained above. PageBlue Protein Staining Solution (Thermo Scientific, 24620) was used to stain for proteins.

### RNase A/T1 treatments

To reproduce recent data concerning RNase sensitivity of glycoRNA^6^, 20 µg of labeled, purified and clicked RNA was mixed with 1 µl RNase A/T1 cocktail (Thermo Scientific), containing 0.5 U RNase A and 20 U RNase T1, in RNase Buffer (20 mM Tris–HCl at pH 8.0, 100 mM KCl and 0.1 mM MgCl_2_). The samples were incubated for 60 min at 37 °C. Afterward, to compare glycan abundance and migration behavior before and after silica column purification, the samples were split in half, where one portion was put on ice and the other was subjected to a silica column purification as explained above. The samples were analyzed by agarose gel electrophoresis and fluorescence in-gel detection. To investigate the importance of intact RNA for the precipitation of glycoproteins in samples of RNA, 16 µg of labeled, purified and clicked RNA was mixed and incubated with RNase A/T1 as stated above. The samples without RNase A/T1 were used as a control. An aliquot equivalent to 2 µg of RNA from each group was separated as an ‘input’ control. Next, the samples were split in three, where each reaction tube was mixed with two volumes of RNA binding buffer (Zymo). Importantly, the RNA binding buffer contains guanidine salts, which inactivate the RNase. The first tube received another volume of isopropanol (100%). The second tube was spiked with 5 µg of intact RNA from 3T3 cells not exposed to Ac_4_ManNAz-labeling and then received another volume of isopropanol (100%). The third tube received two volumes of isopropanol (100%). The column purification proceeded as explained above. The input controls and eluates from the columns were then analyzed via agarose gel electrophoresis.

### Amicon concentration

To probe for impaired column binding of glycosylated molecules following RNase A/T1 treatment, the silica column’s first flow-through (see above) was collected and immediately loaded to an Amicon Ultra centrifugal filter device with a 10-kDa molecular weight cutoff (Milipore, UFC201024) for concentration. The filter membrane was washed three times with 1 ml of ultrapure water and centrifuged at 2,800*g* for 15 min at 4 °C. The concentrated sample was eluted by the inversion of the filter device and centrifugation at 2,800*g* for 1 min, which spun the sample out into a fresh reaction tube. The sample volume was further reduced by vacuum centrifugation. The analysis was conducted by fluorescence in-gel detection.

### Liquid chromatography–mass spectrometry sample preparation and analysis

For sample preparation, total RNA was extracted from 3T3 or HeLa cells with TRIzol and was desalted using silica columns as described above. A portion of this RNA was separated (total RNA). The rest received 1 µg proteinase K per 25 µg RNA, which was directly added to the purified RNA in ultrapure water. After incubation for 45 min at 37 °C, large (>200 nts) and small (<200 nts) RNA fractions were separated as explained above. A total of 100 µg of RNA from each sample group (total RNA, large RNA fraction and small RNA fraction) was digested with 1 µl PNGase F (NEB, P0704S) in 1× GlycoBuffer 2 (NEB) by overnight incubation at 37 °C to remove N-glycans. This was followed by the addition of 1 µl RNase A/T1 cocktail (Thermo Fisher) at 37 °C for 30 min. A short sonication step (30 s, 80% amplitude, 0.5 pulse) was performed to degrade remaining nucleic acids. In total, 100 µl of hot lysis buffer (100 mM ammonium bicarbonate, 2% sodium lauryl sulfate (SLS), 10 mM tris(2-carboxyethyl)phosphine, 95 °C) was added per 100-µl sample, and the samples were boiled for 10 min at 95 °C. Next, 4 mM iodoacetamide was added to the samples and incubated for 30 min, protected from light. A total of 40 µl of Sera-Mag magnetic carboxylate-modified particles (Cytiva) and 40 µl of SpeedBead magnetic carboxylate-modified particles (Cytiva) were mixed, washed three times with Milli-Q water and resuspended in 200 µl of Milli-Q water. In total, 10 µl of this bead suspension was used per sample to capture proteins following the manufacturer’s instructions. On-bead tryptic digestion of proteins was performed by adding 1 µg of sequencing-grade trypsin (Promega) to the beads and overnight incubation at 30 °C and 1,200 rpm. Subsequently, the supernatant was collected, and residual SLS was precipitated by adding 1.5% trifluoroacetic acid and subsequent centrifugation at 17,000*g* for 10 min at 4 °C. The supernatant was separated and further desalted for mass spectrometric analysis using C18 solid phase columns (Chromabond C18 spin columns; Macherey-Nagel). The peptides were dried, reconstituted in 0.1% trifluoroacetic acid and then analyzed using liquid chromatography–mass spectrometry carried out on an Exploris 480 instrument connected to an Vanquish Neo high-performance liquid chromatography and a nanospray flex ion source (all Thermo Scientific). The peptide separation was performed on a reverse phase high-performance liquid chromatography column (75 μm × 42 cm) packed in-house with C18 resin (2.4 μm; Dr. Maisch). The following separating gradient was used: 94% solvent A (0.15% formic acid) and 6% solvent B (99.85% acetonitrile, 0.15% formic acid) to 35% solvent B over 30 min at a flow rate of 300 nl/min. The peptides were ionized at a spray voltage of 2.3 kV, the ion transfer tube temperature was set at 275 °C and 445.12003 *m*/*z* was used as the internal calibrant. The data acquisition mode was set to obtain one high resolution mass spectrometry scan at a resolution of 60,000 full width at half maximum (at *m*/*z* 200) followed by tandem mass spectrometry (MS–MS) scans of the most intense ions within 1 s (cycle 1 s). To increase the efficiency of MS–MS attempts, the charged state screening modus was enabled to exclude unassigned and singly charged ions. The dynamic exclusion duration was set to 14 s. The ion accumulation time was set to 50 ms (mass spectrometry) and 50 ms at 17,500 resolution (MS–MS). The automatic gain control was set to 3 × 10^6^ for mass spectrometry survey scan and 2 × 10^5^ for MS–MS scans. The quadrupole isolation was 1.5 *m*/*z*, and collision was induced with an HCD collision energy of 27%. The mass spectrometry raw data were then analyzed with MaxQuant^26^ and a *Homo sapiens* (Supplementary Data File 1) and a *Mus musculus* (Supplementary Data File 2) uniprot database, respectively (Supplementary Data File). MaxQuant was executed in standard settings without ‘match between runs’ option. The search criteria were set as follows: full tryptic specificity was required (cleavage after lysine or arginine residues); two missed cleavages were allowed; carbamidomethylation of cysteine was set as a fixed modification; and oxidation of methionine and deamidation of asparagine or glutamine were set as variable modification.

### Soluble human LAMP1

For the expression of soluble human LAMP1, the genetic information for LAMP1 amino acids 29–365 (accession NP_005552.3) was amplified from HeLa cDNA with primers sol_hs_LAMP1_f_AfeI: 5′ATATAAGCGCTGCAATGTTTATGGTGAAAAATG and sol_hs_LAMP1_rev_BamHI: 5′ATATGGATCCTCAGTTCTCGTCCAGCAGACAC. The fragment was purified and digested with AfeI and BamHI and cloned into an equivalently treated pcDNA3.1(-) vector originally containing solBDCA2 (accession number OQ817991 https://www.ncbi.nlm.nih.gov/nuccore/OQ817991). The generated construct consists of an N-terminal HLA-A2 signal peptide (MAVMAPRTLLLLLSGALALTQTWAGSHS; P04439.2, amino acids 1–28), a Twin-Strep-tag (WSHPQFEKGGGSGGGSGGSAWSHPQFEKSA) (IBA) followed by LAMP1 amino acids 29–365. The sequence of the LAMP1 fragment was verified by Sanger sequencing. For protein expression, HEK293 cells were transfected using 0.1 µg of the corresponding plasmid and Lipofectamin 2000 in a 96-well format according to the manufacturer’s protocol. The transfected cells were selected and cloned using 0.7 µg/ml G418 (Thermo Fisher Scientific). The purification of secreted proteins from the supernatant was carried out using Strep-Tactin cartridge (IBA) according to the manufacturer’s instructions. The proteins were concentrated after purification with centrifugal filters (10-kDa cutoff, Millipore) and washed to remove the excess of biotin. The final protein concentration was measured using a bicinchoninic acid assay kit (Thermo Fisher Scientific). The proteins were analyzed by SDS–PAGE and subsequent Coomassie staining (see above) or western blot analysis (see below).

### Sol-hLAMP1 purification studies

Sol-hLAMP1 was used as a tool to investigate the possibility of glycoprotein copurification following the published glycoRNA isolation method^6^. To test its phase separation behavior, 2 µg of purified sol-hLAMP1 in 20 µl of ultrapure water was mixed with 1 ml of TRIzol by rigorous pipetting and then incubated and mixed with chloroform as explained above. The aqeuous phase was carefully extracted and mixed with two volumes of isopropanol and then purified using a silica column. To control direct binding to silica columns, 2 µg of sol-hLAMP1 in 100 µl of ultrapure water was mixed with two volumes of RNA binding buffer (Zymo) by vortexing. Next, one, two or three volumes of ethanol or isopropanol (100%) were added, and the samples were vortexed and briefly incubated on ice. The silica column purification continued as explained above. The yields of sol-hLAMP1 were assessed by western blotting (see below). To investigate the influence of intact RNA for adherence of sol-hLAMP1 to silica columns, an experiment identical to the one performed with clicked RNA (‘RNase A/T1 treatments’ section) was conducted using 2 µg of sol-hLAMP1 in 20 µl for each sample. In brief, sol-hLAMP1 was left untreated, treated with 1 µl RNase A/T1 in RNase buffer as explained above, spiked with 5 µg of RNA from 3T3 cells not exposed to Ac_4_ManNAz labeling, or spiked with RNA and additional RNase A/T1. All samples were incubated for 45 min at 37 °C. Aliquots equivalent to 1 µg sol-hLAMP1 were then separated as ‘input’ controls. The rest was mixed with two volumes of RNA binding buffer (Zymo) and another volume of isopropanol (100%). Two additional controls were used: one sample was mixed with RNase A/T1 and then with RNA binding buffer (Zymo) to inactivate the RNase, then received 5 µg of RNA; another sample was mixed with two instead of one volume of isopropanol. All samples were then purified using Zymo Spin IC columns as per the protocol explained above.

### Western blot analysis of sol-hLAMP1 and endogenous LAMP1

Following analysis by SDS–PAGE as explained above, proteins were transferred to polyvinylidene fluoride by semidry western blotting. Polyvinylidene fluoride membranes were blocked overnight in Tris-buffered saline with Tween 20 (TBS-T) containing 5% BSA. Sol-hLAMP1 was detected using Strep-Tactin horseradish peroxidase conjugate (IBA) diluted 1:40,000 (vol/vol) in TBS-T with 5% BSA. The endogenous human LAMP1 was detected using rabbit anti-LAMP1 mAb (Cell Signaling Technology, XP D2D11, 9091) diluted 1:1,000 (vol/vol) in TBS-T with 5% skim milk powder and peroxidase-conjugated goat anti-rabbit IgG (Jackson Immuno Research, 111-035-045) diluted 1:15,000 in TBS-T with 5% skim milk powder as secondary antibody. The actin staining served as a loading control using mouse monoclonal anti-beta-actin antibody (Sigma, A2228) diluted 1:10,000 (vol/vol) in TBS-T with 5% skim milk powder and peroxidase-conjugated anti-mouse IgG (Jackson Immuno Research, 115-035-062) as secondary antibody. The blots were washed three times with TBS-T before and after secondary antibodies were administred. Signals were detected using SuperSignal West Dura (Thermo Scientific, 34075) or Femto (Thermo Scientific, 34096).

## Results

### Labeled glycans are unaffected by RNase A/T1 treatment

**Fig. 1:**
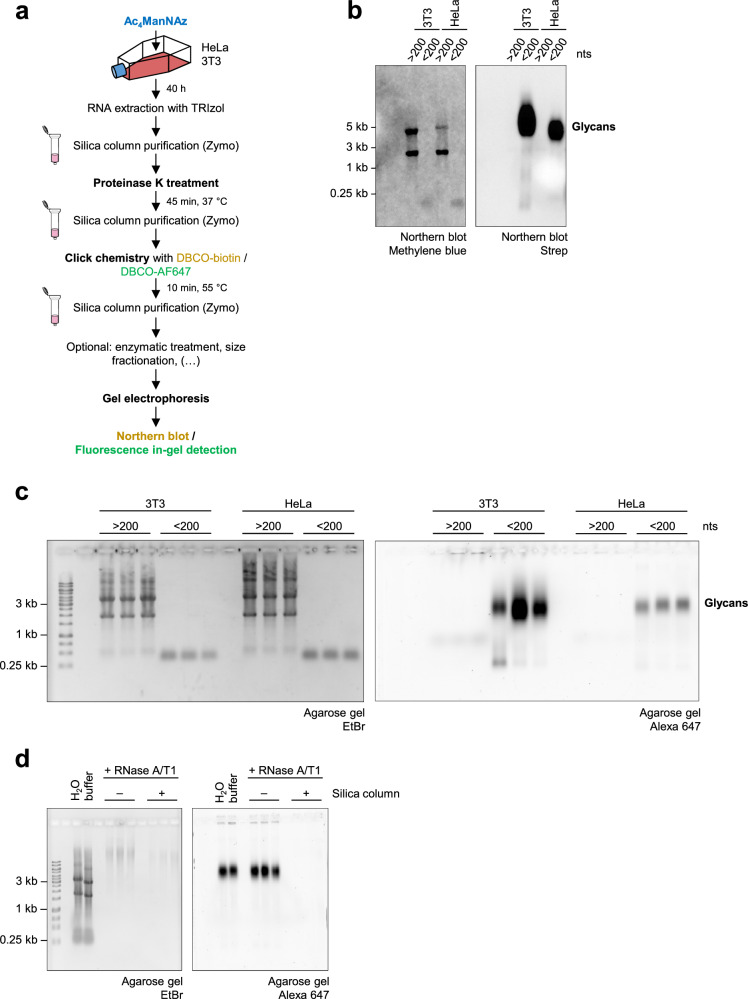
Preparations of glycoRNA contain RNase-resistant glycomolecules. **a** A schematic representation of the glycoRNA labeling and purification strategy, following the procedure described by Flynn et al.^6^. **b** A northern blot of RNA extracted from metabolically labeled 3T3 and HeLa cells. RNA was purified as outlined in **a** and conjugated to DBCO–biotin. Methylene blue was used to detect RNA on the nitrocellulose membrane. A streptavidin–peroxidase probe was used to detect the labeled glycans conjugated to DBCO–biotin. **c** The in-gel fluorescence detection of labeled glycans in preparations of RNA extracted from metabolically labeled 3T3 and HeLa cells. RNA was purified as in **a** and conjugated to DBCO–AF647. Following gel electrophoresis, the labeled glycans were visualized directly in the agarose gel. EtBr staining serves as a loading control. The biological triplicates are shown. **d** The RNase A/T1 treatment of RNA extracted from metabolically labeled HeLa cells. The first two lanes show undigested controls in ultrapure water or in digestion buffer. After the incubation with RNase A/T1, the samples were split. One half was left untreated, and the other was purified using silica columns. Three replicates of individual digestions are shown.

The glycan modification of cellular RNA was investigated following the procedure described by Flynn et al.^6^. The workflow comprises metabolic labeling of cells with Ac_4_ManNAz, RNA extraction with TRIzol^27^, subsequent RNA purification with silica columns, proteinase K treatment and a second silica column purification. To visualize the labeled glycans, RNA samples were subjected to copper-free click chemistry with DBCO–biotin^6^. Large (>200 nts) and small (<200 nts) RNA fractions were separated and subsequently analyzed by northern blotting (Fig. 1a). We observed labeled glycans in small but not large RNA fractions extracted from HeLa and NIH3T3 cells (3T3 cells) (Fig. 1b) in agreement with previous reports^6,13–15^. Although restricted to the small RNA fraction, glycan-modified molecules displayed high apparent molecular weight in the range of 3–5 kb. For a fast and linear workflow that enables the direct detection of labeled glycans in agarose gels, we used copper-free click chemistry to conjugate the fluorophore AF647 to labeled glycans in RNA extracted from metabolically labeled cells. Glycans detected by this in-gel fluorescence detection method displayed high apparent molecular weight and restriction to preparations of small RNA (Fig. 1c), analogous to glycans detected by northern blotting (Fig. 1b), and all subsequent analyses were conducted following this method. To investigate if the glycans detected in RNA preparations were linked to RNA, we subjected DBCO–AF647-clicked RNA from Ac_4_ManNAz-labeled HeLa cells to RNase A/T1 treatment. The labeled glycans were lost after RNase A/T1 treatment and subsequent silica column purification (Fig. 1d) as described by Flynn et al.^6^. However, when RNase A/T1-treated samples were analyzed without postdigestion purification with silica columns, we observed that the migration and integrity of labeled glycans were unaltered (Fig. 1d). This observation implied that glycoRNA samples contained not only glycoRNA but also additional labeled glycosylated molecules that were copurified under the conditions used for glycoRNA isolation.

### Highly stable glycoproteins are present in preparations of glycoRNA

**Fig. 2:**
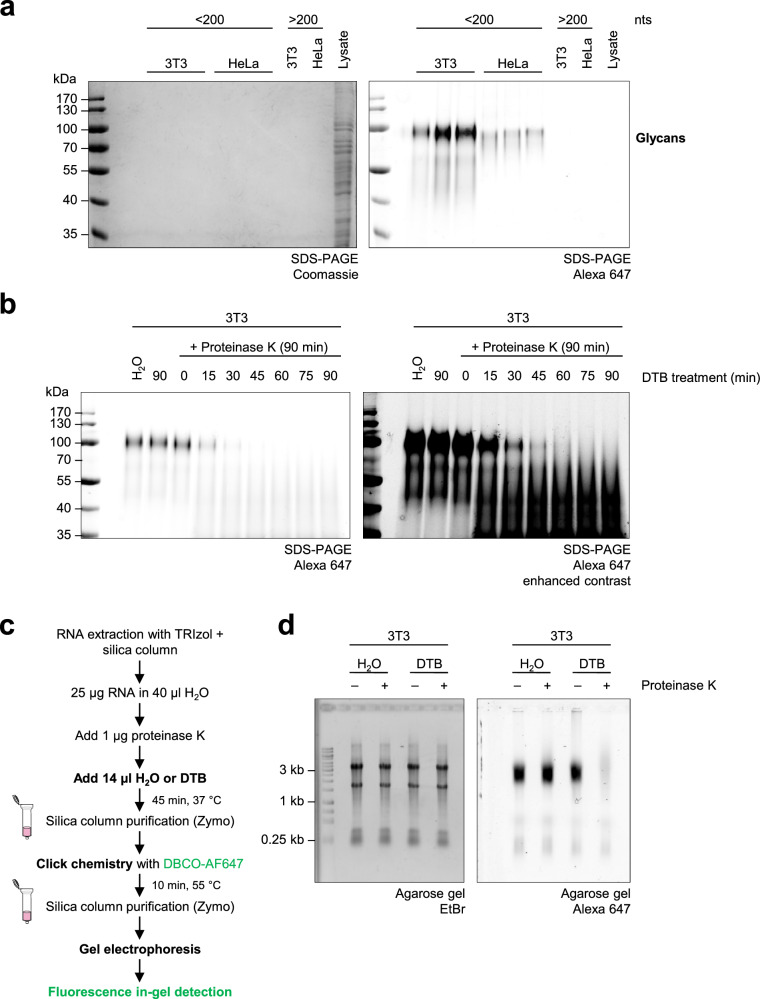
Proteinase K treatment in a denaturing buffer digests the glycosylated molecule. **a** SDS–PAGE of labeled RNAs from Fig. 1c. Left: Coomassie staining was used to detect presence of proteins; cell lysate obtained from native 3T3 cells serves as a positive control. Right: the labeled glycans were visualized by fluorescence in-gel detection. Two samples of ‘large’ RNA (>200 nts) fraction are shown as negative controls. **b** Time-course proteinase K digestion in DTB. In total, 1 µg of proteinase K was directly added to 2 µg of purified RNA in ultrapure water and incubated at 37 °C for 90 min. DTB was added to the RNA–proteinase K mixture in intervals of 15 min as indicated. A representative gel from two independent experiments (*n* = 2) performed in two cell lines is shown (*N* = 4). **c** A schematic representation of a modified proteinase K treatment for glycoRNA purification (Fig. 1a) using DTB for denaturing and reducing conditions. **d** In-gel fluorescence detection of labeled glycans in preparations of RNA extracted from metabolically labeled 3T3 cells, which were subjected to the conventional nondenaturing proteinase K treatment (Fig. 1a) or to the modified proteinase K treatment in DTB (Fig. 2c). The samples without proteinase K in ultrapure water or DTB serve as controls. Left: EtBr staining serves as an RNA loading and integrity control. A representative gel from two independent experiments (*n* = 2) in two cell lines is shown (*N* = 4).

**Fig. 3:**
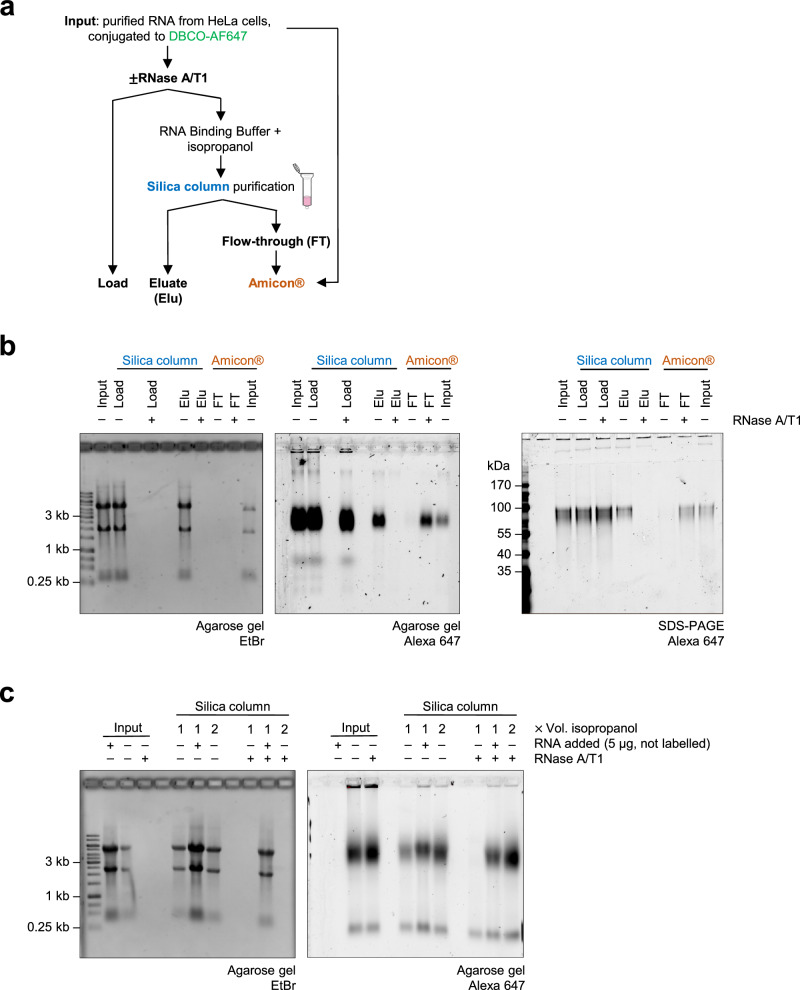
Loss of RNA impairs glycoprotein adherence to silica columns. **a** A schematic representation of a purification strategy to rescue glycosylated molecules from the flow-through of silica columns used for the purification of RNase-treated RNA samples. The flow-through of the first silica column loading step (Material and methods) is saved and transferred to an Amicon concentrator column. **b** In-gel fluorescence detection of Ac_4_ManNAz-labeled glycans rescued from the flow-through of RNase-treated HeLa RNA. The lanes show an input control (‘Input’), samples incubated with or without RNase A/T1 before column loading (‘Load’), after elution (‘Elu’) and the Amicon-concentrated flow-through (‘FT’). An untreated RNA (‘Input’) was subjected to Amicon concentration as a control. Identical samples were analyzed by agarose gel electrophoresis (left and middle panel) and SDS–PAGE (right panel). A representative gel from three independent experiments is shown (*n* = 3). **c** In-gel detection of labeled glycans obtained from silica column purifications in the presence of intact RNA or increased levels of isopropanol. RNA extracted from metabolically labeled 3T3 cells was incubated with or without RNase A/T1. After digestion, samples were split in three and then subjected to a silica column purification with three different conditions during the precipitation step (Material and methods): one tube received one volume of isoproanol, the second received 5 µg of intact RNA and one volume of isopropanol and the third received two volumes of isopropanol. The samples were purified in parallel and analyzed via agarose gel electrophoresis. The samples before the silica column run are shown as controls (‘Input’). A representative gel of two independent experiments is shown (*n* = 2).

To explore if proteins were present in glycoRNA samples, we analyzed small RNA fractions from Ac_4_ManNAz-labeled cells (Fig. 1c) by SDS–PAGE (Fig. 2a). As the samples were digested with proteinase K to eliminate protein contamination^6^, we did not detect protein bands by Coomassie staining as expected. By contrast, we observed several bands with the most prominent signal at approximately 100 kDa by fluorescence in-gel detection (Fig. 2a), possibly implying an insufficient sensitivity of the Coomassie staining. In the original protocol for glycoRNA isolation, proteinase K is directly added to purified RNA in ultrapure water^6^. We speculated that, under these conditions, several proteins might be protected from cleavage owing to inaccessible cleavage sites. Therefore, we treated DBCO–AF647-conjugated RNA extracted from metabolically labeled cells with proteinase K in DTB, containing SDS and 2-mercaptoethanol. We performed proteinase K treatment under denaturing and reducing conditions to enable target protein unfolding and generate access to potentially hidden cleavage sites^22^. Strikingly, proteinase K treatment in DTB led to the degradation of the glycosylated molecule at 100 kDa in a time-dependent manner (Fig. 2b and Supplementary Fig. 1a). No digestion was observed in the absence of proteinase K or when the treatment was conducted in water. Based on these findings, we hypothesized that, using the original protocol^6^, in addition to glycoRNAs, glycoproteins might also be enriched and copurified with the RNA. To test this hypothesis, we isolated RNA from metabolically labeled cells with TRIzol and silica column purification. Purified RNA was digested with proteinase K in DTB or in ultrapure water. Subsequently, the metabolites and buffers were removed by a silica column purification step and samples were clicked to DBCO–AF647 to visualize labeled glycans (Fig. 2c). Significantly, the digestion with proteinase K in DTB but not in water strongly reduced the level of detectable glycans (Fig. 2d and Supplementary Fig. 1b). We did not observe RNA band smearing or loss of ethidium bromide (EtBr) staining, indicating that RNA integrity was not affected by the reaction conditions. These observations highlight that the loss of glycosylated proteins led to a substantial reduction of glycan-modified biomolecules in RNA preparations. To rule out that glycoprotein presence was related to insufficient enzyme concentration during glycoRNA isolation, the experiment was repeated using a 20-fold higher concentration of proteinase K^13,15,20^. No differences in glycomolecule migration or signal intensity were observed when the digestion was performed in water (Supplementary Fig. 1c). By contrast, the glycan signal was strongly reduced for both proteinase K concentrations (1 and 20 µg) when the reaction was conducted in DTB. In light of these findings, we revisited the observation that a postdigestion silica column purification step led to the loss of labeled glycans after RNase treatment (Fig. 1d). We speculated that RNase A/T1 treatment might affect the glycoprotein adherence to silica columns in an indirect fashion. Therefore, we analyzed the flow-through of RNase A/T1-digested HeLa RNA subjected to column purification (Fig. 3a). Importantly, after the concentration using Amicon Ultra columns with a 10-kDa cutoff filter, the glycan-bearing molecules were retrieved from the flow-through showing no signs of degradation when analyzed by agarose gel electrophoresis or SDS–PAGE (Fig. 3b). These results demonstrated that the combination of RNase A/T1 treatment and silica column purification does not induce degradation but rather impairs the column binding ability of glycoproteins that act as contaminants in preparations of glycoRNA. As Kim et al.^20^ recently described that intact RNA and increased concentrations of alcohol benefit the copurification of N-glycosylated molecules when isolating glycoRNA^20^, we spiked RNase A/T1-treated RNA from metabolically labeled cells with intact RNA from cells not exposed to Ac_4_ManNAz-labeling or added an additional volume of isopropanol to the silica column’s precipitation step. Importantly, the loss of labeled glycans after RNase A/T1 digestion and silica column purification could be prevented by both the presence of intact RNA and increased concentrations of isopropanol (Fig. 3c). In summary, our observations suggest that highly stable glycoproteins withstand the purification strategy for glycoRNA. Their copurification is mediated by the presence of intact RNA or high concentrations of alcohol during the column purification step.

### Identification of glycoprotein enrichment in small RNA preparations

**Fig. 4:**
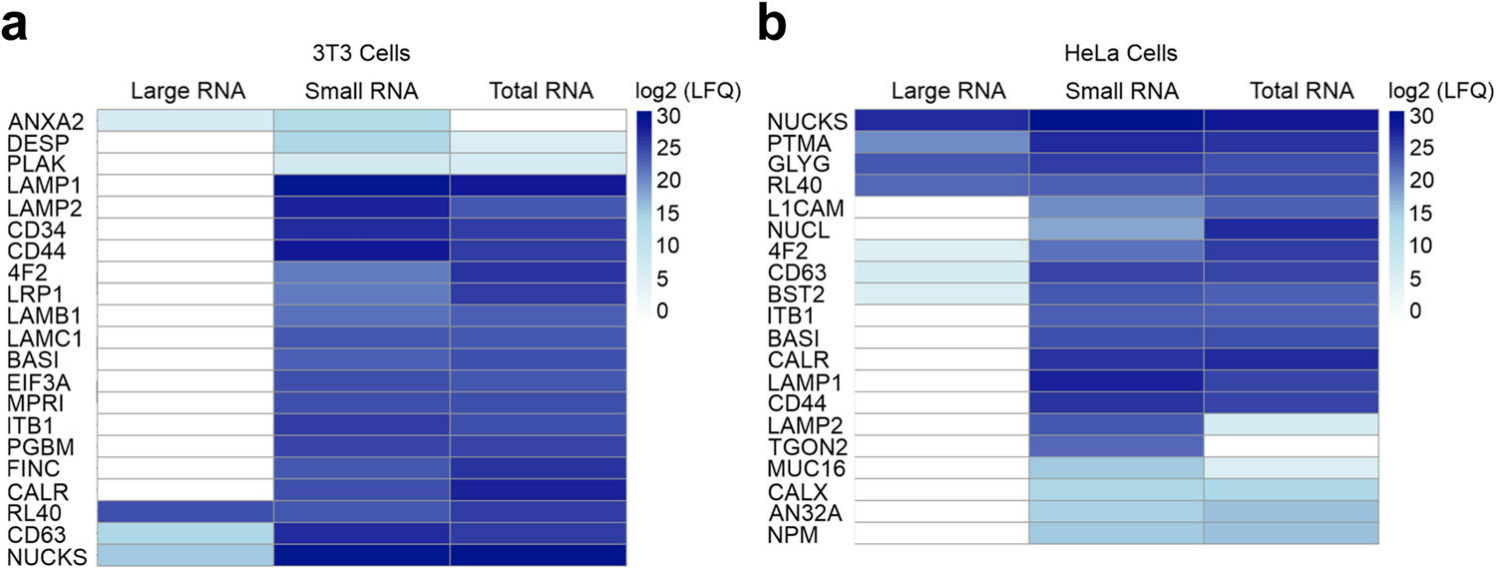
Identification of proteins that copurify with preparations of total, small and large RNA fractions. **a**,**b**, The heat maps display the log_2_-transformed LFQ intensities of identified proteins across large RNA fraction, small RNA fraction and total RNA samples from 3T3 cells (**a**) or HeLa cells (**b**), each analyzed in triplicates. The color scale represents intensity values, with darker shades indicating higher values.

To determine which proteins are copurified with glycoRNAs, we developed a liquid chromatography-MS–MS proteomics workflow. RNA from 3T3 and HeLa cells was isolated and desalted using TRIzol and silica columns as described previously^6^. The RNA was left untreated (total RNA) or was treated with proteinase K, followed by large and small RNA separation^21^ (Supplementary Fig. 2a,b). The samples of all three groups (total RNA, large RNA fraction and small RNA fraction) were digested with PNGase F and RNase A/T1 to remove N-glycans and RNA, respectively, and then subjected to the proteomics pipeline. To ensure confident protein identification, only proteins with an average unique peptide count of ≥2 across small RNA fraction triplicates were taken into consideration (Supplementary Tables 1 and 2). Principal component analysis of sample label-free quantification (LFQ) intensity values revealed the close clustering of replicates from the same sample set for both cell lines (Supplementary Fig. 2c,d). The heatmap illustrating log_2_-transformed LFQ intensities across large RNA fraction, small RNA fraction and total RNA samples isolated from 3T3 cells (Fig. 4a) revealed distinct protein detection profiles. As expected, proteins identified in the small RNA fraction samples were also detected in the total RNA samples, with comparable intensities. By contrast, the majority of proteins detected in the small RNA fraction and total RNA samples were absent in the large RNA fraction samples. The heatmap of log_2_-transformed LFQ protein intensities in large RNA fraction, small RNA fraction and total RNA samples isolated from HeLa cells (Fig. 4b) exhibits a similar protein detection profile to the samples of 3T3 cells. Importantly, ten proteins were commonly detected in the small RNA fractions of both HeLa and 3T3 cells, namely LAMP1, LAMP2, ITB1, 4F2, CALR, CD44, CD63, NUCKS, RL40 and BASI (Supplementary Fig. 2e,f). It is important to mention that RL40, CD63 and NUCKS were also detected in the large RNA fraction samples isolated from 3T3 cells. Similarly, RL40, NUCKS, 4F2 and CD63 were present in the large RNA fraction samples isolated from HeLa cells. Taken together, these data suggest that specific proteins copurify within preparations of proteinase K-treated small RNA fractions.

### Recombinant LAMP1 can be purified using TRIzol and silica columns

**Fig. 5:**
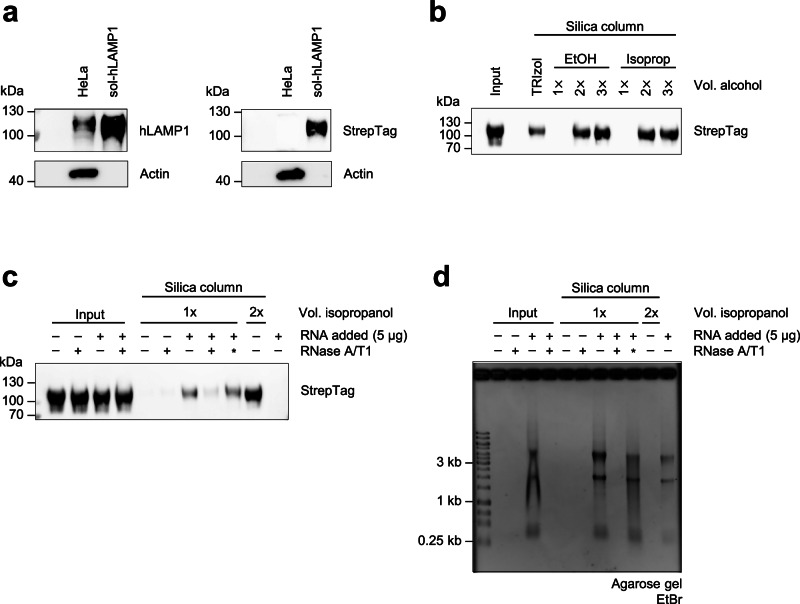
sol-hLAMP1 can be extracted from TRIzol and binds to silica columns. **a** Western blot analysis of the purified sol-hLAMP1 contruct compared with the endogenous LAMP1 obtained from a HeLa cell lysate. **b** Western blot analysis of sol-hLAMP1 extracted via TRIzol or bound directly to a silica column. For the direct column loading, the samples were mixed with the silica column’s RNA binding buffer (Zymo) and additional volumes of ethanol or isopropanol as indicated. In the case of the TRIzol treatment, sol-hLAMP1 was extracted from the aqeuous phase and mixed with two volumes of isopropanol. All samples were then purified using silica columns according to the manufacturer’s protocol and analyzed by western blotting. A representative blot of two independent experiments (*n* = 2) is shown. **c** A western blot analysis of sol-hLAMP1 obtained from silica column purifications in the presence of intact RNA or increased levels of isopropanol, analogous to Fig. 3c. Sol-hLAMP1 was mixed with RNA and/or RNase A/T1 and incubated at 37 °C for 45 min. The aliquots were separated (‘Input’), and the rest was mixed with RNA binding buffer (Zymo) and isopropanol as indicated. The asterisk indicates the inactivation of RNase A/T1 by guanidine salts before adding RNA. The silica column purification was carried out following the manfacturer’s protocol. A representative blot of two independent experiments (*n* = 2) is shown. **d** The agarose gel with EtBr staining of the samples shown in **c**.

Given the identification of LAMP1 in the proteomics data (Fig. 4), and its description as a glycosylated membrane protein^28–30^, we studied the copurification of LAMP1 under the conditions of the glycoRNA purification protocol^6^. We generated a recombinant Twin-StrepTag-containing soluble human LAMP1 (sol-hLAMP1), which showed similar glycosylation compared to endogenous LAMP1 as indicated by the mass increase to more than 100 kDa (Fig. 5a). First, we investigated the phase separation of sol-hLAMP1 during TRIzol extraction and its ability to bind the silica columns used for RNA purification. To this end, sol-hLAMP1 was mixed with TRIzol, the aqueous phase was separated and two volumes of isopropanol were added. Alternatively, sol-hLAMP1 was directly loaded to the silica columns in the presence of one, two or three volumes of alcohol to test for the influence of increased alcohol concentrations for sol-hLAMP1 recovery. Remarkably, sol-hLAMP1 was readily detectable after extraction with TRIzol (Fig. 5b), indicating that sol-hLAMP1 showed hydrophilic phase separation behavior. Interestingly, one volume of either ethanol or isopropanol did not support its recovery. By contrast, two and three volumes of ethanol or isopropanol enabled the efficient sol-hLAMP1 purification. To investigate if the presence of RNA promotes the purification of sol-hLAMP1, similar to experiments conducted by Kim et al.^20^ (and Fig. 3c), we spiked sol-hLAMP1 with native RNA and performed a silica column purification using one volume of isopropanol for precipitation. RNase A/T1 treatment was used as a control. Before the column purification, neither RNase A/T1 treatment nor the addition of RNA affected the detectability of sol-hLAMP1 (Fig. 5c, d). After the column purification, untreated or RNase A/T1-treated sol-hLAMP1 was no longer detectable. By contrast, sol-hLAMP1 could be recovered upon the addition of RNA, which could be reverted by RNase A/T1 treatment. The inactivation of RNase A/T1 rescued sol-hLAMP1. As expected, the addition of two volumes of isopropanol facilitated the most efficient recovery. Taken together, our results indicate that sol-hLAMP1 as a representative glycoprotein has the physicochemical properties that allow copurification during extraction of glycoRNA^6^. In addition, the presence of intact RNA or high alcohol concentrations are factors that enhance the copurification of glycoproteins via silica columns.

## Discussion

Metabolic labeling is one of the core methods that enabled the identification and analysis of glycoproteins^7,31,32^. Flynn et al. used metabolic labeling with bioorthogonal sugar analogs to demonstrate that also RNA can be modified with glycans^6^, creating the emerging field of glycoRNA^6,14–19^. One key property of glycoRNA is its sensitivity toward RNase treatment as reported before^6^. However, we observed that glycoRNA preparations contain additional RNase-resistant glycomolecules, whose loss after RNase treatment depends on a subsequent silica column purification step. In line with findings by Kim et al., we demonstrate that these glycomolecules can adhere to silica columns in an RNA- and alcohol-concentration-dependent manner^20^. Combining the observations that proteinase K digestion under denaturing conditions led to the degradation of these molecules and the identification of 20 and 21 proteins in small RNA fractions extracted from 3T3 and HeLa cells, respectively, we conclude that proteins may copurify with RNA under the original glycoRNA purification protocol^6^.

The presence of proteins in samples of proteinase K-treated small RNA preparations suggests that these proteins possess three essential chemical properties that enable their copurification and protection during glycoRNA isolation^6^: (1) hydrophilic phase separation during organic phenol-chloroform extraction, (2) binding to silica columns and (3) relative resistance to proteinase K treatment under nondenaturing conditions. Significantly, most proteins identified here are known either to be glycosylated or to possess RNA-binding capabilities, often comprising both features (Supplementary Tables 1 and 2), suggesting that these factors facilitate copurification. Whereas ten proteins were commonly identified between 3T3 and HeLa cells (Supplementary Fig. 2e,f), the identification of individual hits, unique for either 3T3 or HeLa cells, is probably related to cell type- and species-specific expression and modification patterns. To study the possibility of glycoprotein copurification during RNA extraction, we used LAMP1 as a representative glycoprotein from the group of commonly identified proteins. We demonstrated that purified sol-hLAMP1 at least partially localized to the aqueous phase during TRIzol extraction (Fig. 5b), which is probably mediated by its extensive decoration with glycans^28,29^, which can significantly enhance hydrophilicity^2^. Indeed, it is established that, along with association to RNA, glycan modification can disturb ‘canonical’ phase separation^33^. For instance, the contamination of RNA and DNA samples, extracted from blood or tissue, with the glycosaminoglycan heparin is a known phenomenon^34,35^.

We observed that the recovery of glycoproteins from silica columns was dependent on the presence of RNA and high alcohol concentrations (Figs. 3 and 5). These observations stand in line with findings by Kim et al., who demonstrated that RNA acts as a coprecipitant for an N-glycosylated molecule, reducing the minimal alcohol concentration required for its binding to the silica resin. The digestion of RNA by RNase treatment impaired the N-glycosylated molecule’s adherence to the silica surface, thereby mimicking an RNase-sensitive molecule. However, at high alcohol concentrations (that is, increasingly nonpolar solvents), RNA became negligible for the recovery^20^. In our proteomics approach (Fig. 4), large and small RNAs were separated on the basis of the alcohol concentration during column loading (using high alcohol concentrations for small RNAs), leading to a strong overrepresentation of proteins in the small RNA fraction. In addition, we observed that sol-hLAMP1 was recovered from silica columns depending on the presence of RNA or higher concentrations of alcohol (Fig. 5). Thus, we conclude that the mechanisms described by Kim et al. also apply to glycoproteins. Besides this partial dependency on RNA, protein composition is probably another important factor that determines the affinity to silica resins in the present work. Proteins with high contents of charged amino acids, unstructured regions and RNA-binding motifs are known to bind to silica nanoparticles in cell extracts^36,37^. Mechanistically, protein charge is involved in the initial binding phase, whereas protein flexibility via intrinsic disorder mediates spreading across the silica surface and leads to tighter adherence. In the case of our model glycoprotein LAMP1, its arginine-rich cytosolic tail was recently found to bind RNA^38^, and its hinge region contains an intrinsically disordered region, thus fulfilling several of the criteria that could mediate silica coating. However, intrinsically disordered regions can also exert nonclassical modes of RNA-binding as seen in condensates^39^, membrane-less organelles that are known to incorporate RNA^40^. Thus, it is possible that physical binding to RNA also promotes the copurification of glycoproteins such as LAMP1 during the purification of glycoRNA following the published protocol^6^.

Regarding resistance to proteinase K, extensive glycosylation can protect from proteolytic cleavage either by shielding cleavage sites or via electrostatic repulsion^2^. For example, O-glycosylated fragments of mucins were found to be largely resistant to proteinase K cleavage, a property that has been exploited to isolate these from other proteins that are more susceptible to digestion^41,42^. In the case of LAMP1, relative protection from proteinase K is expected, given that the glycomatrix of LAMP1^28,29^ helps to protect the endosome from autolysis^43,44^. Although proteinase K is active across a wide range of buffers and pH^45^, its activity can be stimulated upon the addition of denaturing and reducing agents, which generate access to hidden cleavage sites in heavily structured target proteins^22^. We observed that the use of a buffer system with denaturing and reducing activity during proteinase K digestion leads to the removal of glycoprotein contaminants from glycoRNA preparations (Fig. 2). By contrast, a 20-fold higher enzyme concentration did not visibly affect the presence of glycomolecules when the treatment was conducted in water (Supplementary Fig. 1c), emphasizing that the reaction conditions are a more critical aspect for glycoprotein removal than the enzyme concentration.

The extractability of glycoproteins by TRIzol, their resistance to proteinase K under nondenaturing conditions and their quasi RNA-dependent binding to silica columns can create the impression of an RNase-sensitive molecule and, thus, glycoRNA. As high nuclease resistance of glycosylated molecules in RNA preparations was reported by other studies before^15,20,46^, we hypothesize that the copurification of glycoproteins during glycoRNA isolation is a common phenomenon. The denaturing proteinase K treatment we described here may serve as a useful tool to fine-tune the established method toward higher glycoRNA sample purity. It is important to note that our studies are confined to samples generated by metabolic labeling with Ac_4_ManNAz, which primarily labels N-linked sialoglycans in RNA preparations^6^. It is possible that O-glycosylated peptides, which were not efficiently labeled by Ac_4_ManNAz and thus not detectable, remain present in the samples. Indeed, several of the proteins we identified (Fig. 4) are known to be O-glycosylated (Supplementary Tables 1 and 2). Current advances in the field of glycoRNA research include labeling strategies that target not only N-linked but also O-linked sialoglycans, such as the recently developed RNA-optimized periodate oxidation and aldehyde ligation (rPAL)^13^, which enabled the discovery of O-linked glycoRNAs^47^. Likewise, galactose- or glucose-based azido-sugars may also preferentially label O-glycans and were recently used to detect potential subspecies of glycoRNAs^46,48^. The implementation of additional digestion steps that ensure removal of both N- and O-glycosylated proteins, as well as nonglycosylated protease-resistant proteins, will be important for future studies. Current rPAL-based protocols already use the mucinase StcE as a second digestion step after proteinase K to remove contaminating mucins, an important source of O-glycans^13^. The combination of denaturing proteinase K treatment and mucinase StcE may increase the specificity of the purification method for glycoRNAs and be further optimized by future investigations. Otherwise, RNA isolation under conditions with no or mild proteolysis may enrich for glycosylated and/or RNA-binding proteins.

In summary, we here describe the copurification of glycoproteins during the preparation of glycoRNA following the recently described method^6^. We hypothesize that the glycosylation status and RNA-binding properties of the proteins identified herein mediate both their copurification and protection during isolation. Further insights into the complex nature of glycoRNA are needed. The considerable presence of glycoproteins in RNA preparations suggests that a strong part of the signal originally attributed to glycoRNA is protein-derived. Such glycoproteins can complicate the biochemical analyses of structural and functional aspects of glycoRNA. Our studies warrant caution when isolating glycoRNA and interpreting functional aspects related to this modification and will guide future efforts to refine techniques for the reliable discrimination of glycosylated biomolecules.

## Supplementary information

Supplementary Information

Supplementary Data File 1

Supplementary Data File 2
