# Supplementary material for: Proteins are a source of glycans found in preparations of glycoRNA
Source: Exp Mol Med. 2025 Nov 14;57(11):2505–16. doi: 10.1038/s12276-025-01575-1 (PMC12685969; doi:10.1038/s12276-025-01575-1)
Supplement: Supplementary file 1 — Supplementary Information [file 12276_2025_1575_MOESM1_ESM.pdf]

Supplementary information for the manuscript entitled

Proteins are a source of glycans found in preparations of glycoRNA

Nathanael B. Kegel[a], Nurseda Yilmaz Demirel[b], Timo Glatter[b], Katharina Höfer[b,c,d], Andreas Kaufmann[a] & Stefan Bauer[a]

Affiliations

[a] Institute of Immunology, Philipps-Universität Marburg, Marburg, Germany

[b] Max-Planck-Institute for Terrestrial Microbiology, Marburg, Germany

[c] Center for Synthetic Microbiology (SYNMIKRO), Philipps-Universität Marburg, Marburg, Germany

[d] Department of Pharmacy, Institute of Pharmaceutical Biology and Biotechnology, Philipps-Universität Marburg, Marburg, Germany

Supplementary Figure 1

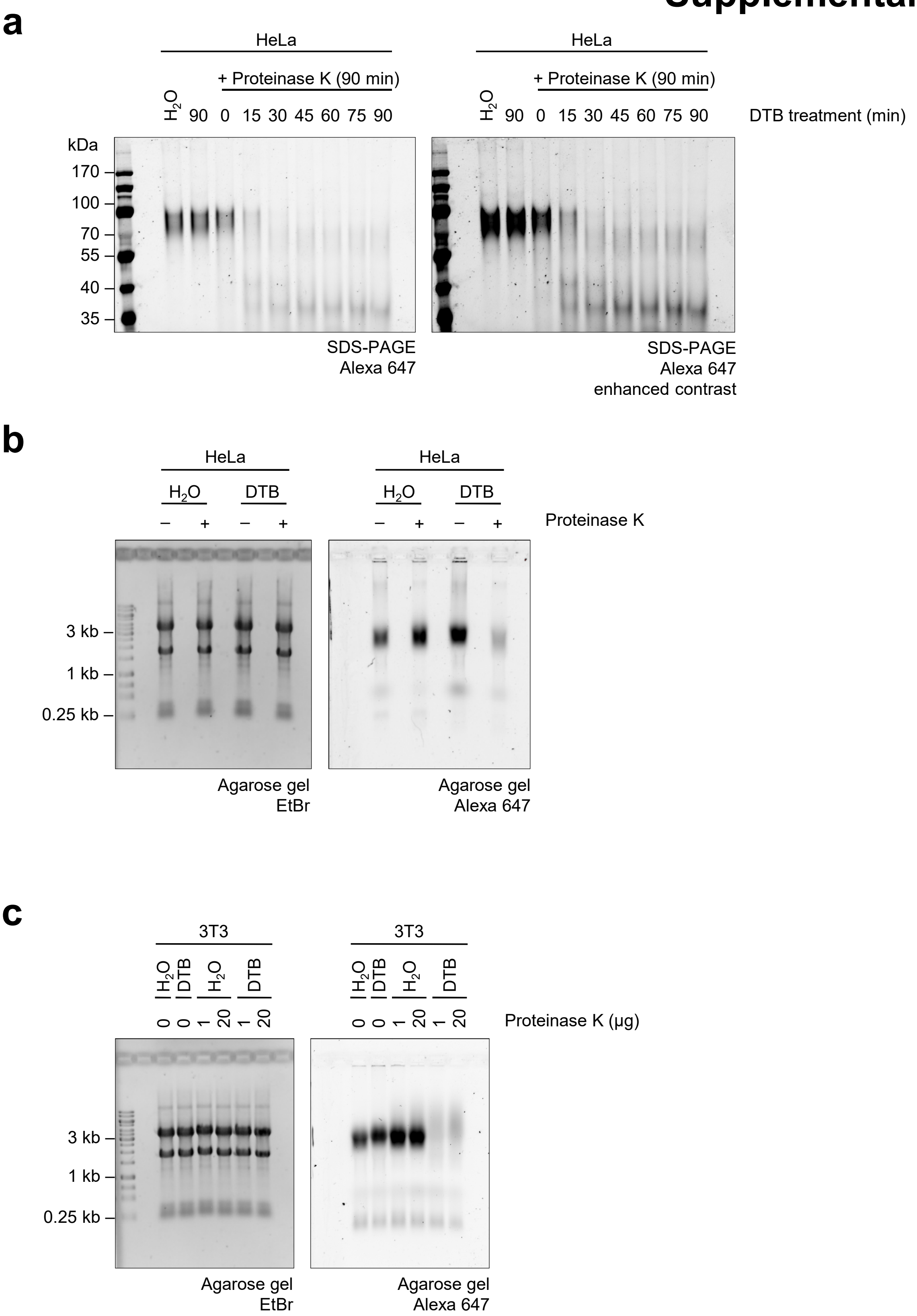

Supplementary Fig. 1, related to Figure 2: Proteinase K treatment in denaturing Tris buffer using HeLa RNA as a substrate or using 20-fold more enzyme. Experiments in (a) and (b) were conducted as in Figure 2b and 2d using DBCO-AF647-clicked RNA from metabolically labelled HeLa cells. The experiment in (c) was conducted as in Figure 2d using 1 or 20 µg proteinase K per 25 µg RNA after TRIzol extraction. The treatment was conducted in water or DTB. Samples without proteinase K were used as controls.

Supplementary Figure 2

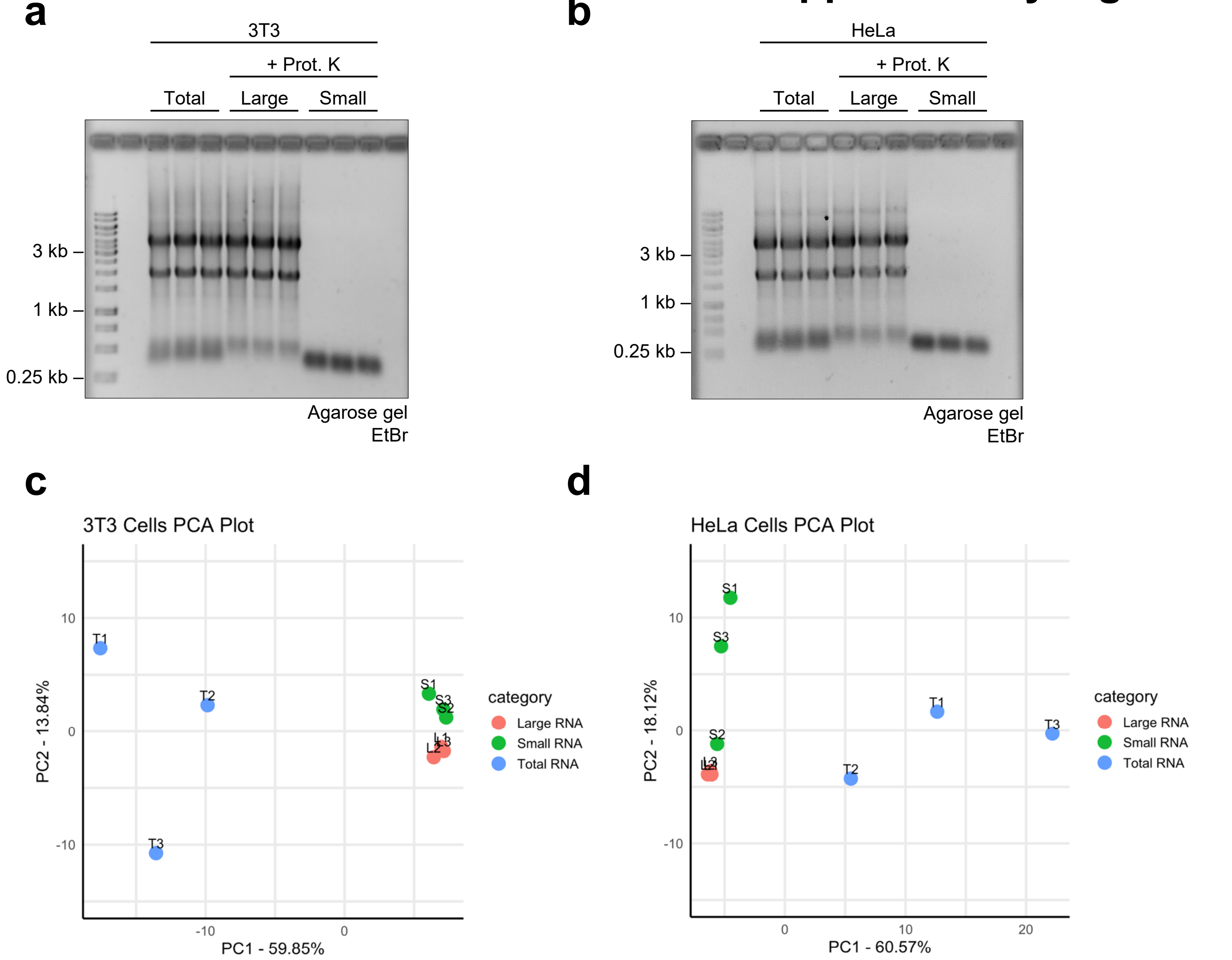

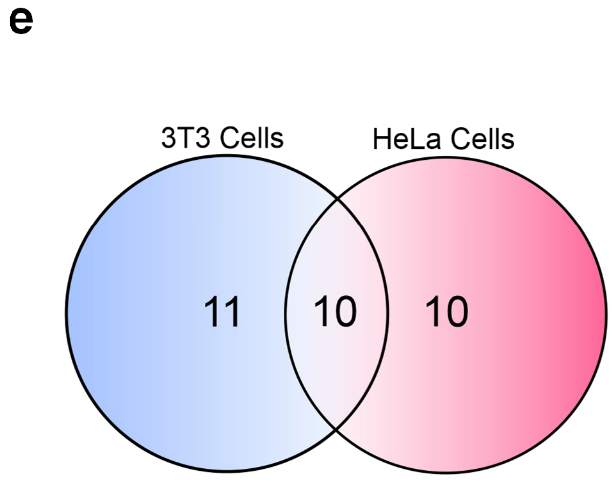

f

The List of Commonly Detected Proteins
LAMP1
LAMP2
ITB1
4F2
CALR
CD44
CD63
NUCKS
BASI
RL40

Supplementary Fig. 2, related to Figure 4: RNA samples for proteomics analysis, PCA results, and commonly detected proteins. (a) Agarose gel with EtBr staining of the three sample groups (total RNA, large RNA fraction, small RNA fraction) extracted and purified from 3T3 cells. (b) Agarose gel as in (a) but with samples obtained from HeLa cells. (c) Principal Component Analysis (PCA) of sample label-free quantification (LFQ) intensities to evaluate the reproducibility of triplicates. The score plot illustrates the distribution of individual samples along the first two principal components

(PC1 and PC2). PCA of samples from 3T3 cells showing the close clustering of replicates from the same sample set. (d) PCA of samples from HeLa cells showing the close clustering of replicates from the same sample set. (e) Venn diagram illustrating the number of individually detected proteins as well as commonly detected proteins between 3T3 and HeLa cells. (f) Table displaying the list of commonly detected proteins.

Supplementary Tables

Supplementary Table 1: Proteins with an average unique peptide count of ≥2 identified in small RNA fraction samples extracted from 3T3 cells with information on molecular weight, glycosylation, and RNA-binding properties.

**Protein IDs**<br><br>Uniprot accession number	**aa / MW (kDa)**	**Glycosylation sites reported on UniProt**<br><br>including inferred sites based on sequence analysis	**Known interaction with RNA**<br><br>including occurrences in high-throughput analyses
**ANXA2**<br>P07356	339 / 38.7	None, but probably glycosylated in humans (PMID: 1445299)	No
**DESP**<br>E9Q557	2883 / 332.9	None	No
**PLAK**<br>Q02257	745 / 81.8	1 O-linked (sequence analysis, PMID: 12847106)	Yes (PMID: 27281784, 29339797)
**LAMP1**<br>P11438	406 / 43.9	18 N-linked (PMID: 27663661, 16170054, 19349973, 19656770, 16944957)	Yes (PMID: 25772617)
**LAMP2**<br>P17047	415 / 45.7	16 N-linked (PMID: 2766366)	Yes (PMID: 23291500)
**CD34**<br>Q64314	382 / 41.0	7 N-linked (PMID: 19656770)	No
**CD44**<br>P15379	778 / 85.6	10 N-linked (sequence analysis)<br>1 O-linked (by similarity, see human CD44)	No
**4F2**<br>P10852	526 / 58.3	8 N-linked (PMID: 19349973, 19656770)	Yes (PMID: 21266579)
**LPR1**<br>Q91ZX7	4545 / 504.7	51 N-linked (PMID: 19656770, 19349973)	No
**LAMB1**<br>P02469	1786 / 197.1	12 N-linked (PMID: 19349973)	No
**LAMC1**<br>P02468	1607 / 177.3	14 N-linked (PMID: 19349973)	No

**BASI**<br>P18572	389 / 42.4	3 N-linked (PMID: 19349973, 19656770)	No
**EIF3A**<br>P23116	1344 / 161.9	O-glycosylated (PMID: 34887587)	Yes (PMID: 17581632)
**MPRI**<br>Q07113	2483 / 273.8	20 N-linked (PMID: 19349973, 19656770)	No
**ITB1**<br>P09055	798 / 88.2	12 N-linked (PMID: 19656770)	No
**PGBM**<br>Q05793	3707 / 398.3	4 O-linked (sequence analysis + by similarity)<br>10 N-linked (PMID: 19656770, 19349973)	No
**FINC**<br>P11276	2477 / 272.5	8 N-linked (PMID: 17330941, 19656770, 16944957)	No
**CALR**<br>P14211	416 / 48.0	1 N-linked (see human CALR)<br><br>binds to all monoglycosylated proteins in the Golgi apparatus (PMID: 20880849, 21652723)	Yes (in humans; PMID: 12242300)
**RL40**<br>P62984	128 / 14.7	None	Part of the 60S ribosomal subunit (PMID: 19754430, 36517592)
**CD63**<br>P41731	238 / 25.8	4 N-linked (PMID: 19349973)	No
**NUCKS**<br>Q80XU3	234 / 26.3	None	Yes (in humans: PMID: 22681889)

Supplementary Table 2: Proteins with an average unique peptide count of ≥2 identified in small RNA fraction samples extracted from HeLa cells with information on molecular weight, glycosylation, and RNA-binding properties.

**Protein IDs**<br><br>Uniprot accession number	**aa / MW (kDa)**	**Glycosylation sites reported on UniProt**<br><br>including inferred sites based on sequence analysis	**Known interaction with RNA**<br><br>including occurrences in high-throughput analyses
**NUCKS**<br>Q9H1E3	243 / 27.3	None	Yes (PMID: 22681889)
**PTMA**<br>P06454	111 / 12.2	None	Yes (PMID: 2452757, 2479575, 9326492)
**GLYG**<br>P46976	350 / 39.4	1 O-linked (PMID: 22160680, 30356213)	No
**RL40**<br>P62987	128 / 14.7	None	Is a component of the 60S ribosomal subunit (PMID: 23169626, 23636399, 32669547, 39048817, 39103523)
**L1CAM**<br>P32004	1257 / 140.0	20 N-linked (PMID: 16335952 , 19159218)	No
**NUCL**<br>P19338	710 / 76.6	A surface-located form of NUCL is glycosylated (PMID: 21575138, 15823039, 19023103, 19026635	Yes (PMID: 8321232, 16213212)
**4F2**<br>P08195	630 / 68.0	4 N-linked (PMID: 12754519, 19159218, 19349973, 30867591, 33298890, 33758168, 34880232, 35352032, 16335952, 19139490)	Yes (PMID: 21266579, 22658674)
**CD63**<br>P08962	238 / 25.6	3 N-linked (PMID: 12754519, 19159218)	No
**BST2**<br>Q10589	180 / 19.8	2 N-linked (PMID: 19159218, 19349973, 19737401, 19879838)	Yes (PMID: 22658674)
**ITB1**<br>P05556	798 / 88.4	12 N-linked (PMID: 19159218, 22451694, 33962943, 16335952, 19349973)	No

**BASI**<br>P35613	385 / 42.2	3 N-linked (PMID: 12754519, 19159218, 19349973)	No
**CALR**<br>P27797	417 / 48.1	1 N-linked (PMID: 19159218)<br>binds to all monoglycosylated proteins in the ER (PMID: 7876246)	Yes (PMID: 14726956, 22658674)
**LAMP1**<br>P11279	417 / 44.9	18 N-linked (PMID: 3143719, 12754519, 19159218, 16335952)<br><br>6 O-linked (PMID: 8323299	Yes (PMID: 25772617)
**CD44**<br>P16070	742 / 81.5	9 N-linked (PMID: 12883358, 16335952, 19159218, 22171320)<br><br>1 O-linked (PMID: 25326458, 32337544, 36213313, 37453717)	No
**LAMP2**<br>P13473	410 / 45.0	16 N-linked (PMID: 2243102, 2912382, 8323299, 12754519, 16335952, 19159218)<br><br>10 O-linked (PMID: 8323299)	Yes (PMID: 23291500)
**TGON2**<br>O43493	437 / 45.9	9 N-linked (sequence analysis)	No
**MUC16**<br>Q8WXI7	14507 / >1500	102 N-linked, heavily O-glycosylated (PMID: 12734200),	No
**CALX**<br>P27824	592 / 67.6	None, but functions as a chaperone during glycosylation quality control in the ER (PMID: 8203019, 7736594)	Yes (PMID: 22658674, 22681889)
**AN32A**<br>P39687	249 / 28.6	None	Yes (PMID: 22681889)
**NPM**<br>P06748	294 / 32.6	None	Yes (PMID: 12058066, 24106084)
